# Supplementary material for: Components of Perinatal Palliative Care: An Integrative Review
Source: Children (Basel). 2023 Mar 1;10(3):482. doi: 10.3390/children10030482 (PMC10047326; doi:10.3390/children10030482)
Supplement: Supplementary file 1 [file children-10-00482-s001.zip › Suppl S2 Search strategy.pdf]

Supplementary information 2: Detailed search strategy

The search strategy was structured around two distinct blocks: population and palliative care, based on the concepts and definitions provided in Box 2. Additionally, the block ‘perinatal palliative care’ was added in case the combination of the two individual search blocks overlooked relevant research.

The MEDLINE syntax was used as a starting point for the construction and validation of the search strategy. For construction of the search strategy, the search blocks was based on previous search filters as well as Medical Subject Headings (MesH) terms and free text words. Relevant papers resulting from a scoping review were consulted for MesH terms and relevant free text words to set up and/or refine the search strategy. An information specialist specialized in the development of search strategies, database searches and systematic review of literature was consulted throughout this process.

To test the reliability and the sensitivity of this search strategy, a scoping review was done. A set of records used to validate the search strategy was constructed by hand-searching volumes of the five journals that provided the highest number of relevant articles during this initial scoping review in the period between 1997 and 2021. This set of records included all articles resulting from this hand-search that adhered to our eligibility criteria.

The MEDLINE search strategy was translated to the other databases.
